# A quantitative approach for measuring laterality in clinical fMRI for preoperative language mapping

**DOI:** 10.1007/s00234-021-02685-z

**Published:** 2021-03-26

**Authors:** Maria Olaru, Ryan M. Nillo, Pratik Mukherjee, Leo P. Sugrue

**Affiliations:** Department of Radiology and Biomedical Imaging, University of California, San Francisco, San Francisco, CA USA

**Keywords:** Task fMRI, Language fMRI meta-analysis, Laterality index, Pre-operative language mapping

## Abstract

**Purpose:**

fMRI is increasingly used for presurgical language mapping, but lack of standard methodology has made it difficult to combine/compare data across institutions or determine the relative efficacy of different approaches. Here, we describe a quantitative analytic framework for determining language laterality in clinical fMRI that addresses these concerns.

**Methods:**

We retrospectively analyzed fMRI data from 59 patients who underwent presurgical language mapping at our institution with identical imaging and behavioral protocols. First, we compared the efficacy of different regional masks in capturing language activations. Then, we systematically explored how laterality indices (LIs) computed from these masks vary as a function of task and activation threshold. Finally, we determined the percentile threshold that maximized the correlation between the results of our LI approach and the laterality assessments from the original clinical radiology reports.

**Results:**

First, we found that a regional mask derived from a meta-analysis of the fMRI literature better captured language task activations than masks based on anatomically defined language areas. Then, we showed that an LI approach based on this functional mask and percentile thresholding of subject activation can quantify the relative ability of different language tasks to lateralize language function at the population level. Finally, we determined that the 92nd percentile of subject-level activation provides the optimal LI threshold with which to reproduce the original clinical reports.

**Conclusion:**

A quantitative framework for determining language laterality that uses a functionally-derived language mask and percentile thresholding of subject activation can combine/compare results across tasks and patients and reproduce clinical assessments of language laterality.

**Supplementary Information:**

The online version contains supplementary material available at 10.1007/s00234-021-02685-z.

## Introduction

Many institutions have replaced traditional Wada testing with clinical fMRI for presurgical language mapping [1]. Supporting this trend, the American Academy of Neurology recently judged the two techniques to be equivalent for preoperative assessment of language laterality in epilepsy patients [2]. However, the Academy also emphasized the lack of evidence that fMRI can predict postoperative language outcomes or localize language beyond the hemispheric level. Efforts to apply clinical fMRI to these more nuanced questions are hindered by a lack of standard methodology in the field, which makes it difficult to combine data across institutions or determine the relative efficacy of different approaches. Indeed, with increasing recognition that language networks are more complex than suggested by the classical model of anterior-expressive (Broca’s) and posterior-receptive (Wernicke’s) areas, even the set of candidate regions to consider in localizing language is unclear [3–5].

Addressing these issues, the American Society of Functional Neuroradiology has recommended that the field adopt standard tasks and approaches [6]; however, these standards are currently largely based on consensus opinion rather than the systematic and quantitative comparison of approaches and data across tasks, patients, and institutions. As Bradshaw and colleagues detailed in a recent comprehensive review, quantitative methods for language lateralization with fMRI will need to address the key elements of thresholding, regions of interest, and tasks [7]. Here, we develop such a quantitative approach and use it to compare the relative efficacy of a limited set of language mapping tasks in a population of 59 patients who underwent presurgical fMRI language mapping at our institution.

Our approach differs from previously published work in 4 respects: (1) An inclusive clinical cohort representative of patients who undergo preoperative fMRI for assessment of language laterality. (2) Language regions of interest derived from a meta-analysis of the published fMRI language literature. (3) A whole brain normalization method that is robust to outliers and variability in distributions of activation between subjects and which allows for population-level analysis, and (4) Validation of the approach through independent subject- and group-level analyses. We aim to create an automated framework for quantifying language laterality in preoperative clinical fMRI that can: (1) perform at the level of current clinical practice without input from highly trained clinicians, and (2) facilitate the collection of standardized data for the purpose of establishing best practices and contributing to our understanding of human language processing.

## Materials and methods

### Study population

With institutional review board approval, we searched our institution’s database for cases of clinical fMRI for presurgical language mapping between January 2011 and December 2018 (*N* = 144). Informed consent was not required due to the retrospective nature of the study. The resulting retrospective, cross-sectional dataset comprised all patients who met the following inclusion criteria: (1) testing with identical behavioral and imaging protocols on the same 3T scanner and (2) completion of all 4 tasks. We excluded patients whose data were deemed non-interpretable or technically inadequate at the time of original testing. Examples of reasons provided in the reports for non-interpretable studies included “inability to cooperate,” “difficulty with interpretation due to motion,” or “limited reading ability (in English).” Based on these criteria, 66 patients were excluded because they did not complete all 4 fMRI tasks, and 19 additional patients were excluded because their data were described as non-interpretable in the original radiology report, often because of patient motion/inability to perform tasks or due to the presence of large lesions that made results unreliable due to mass effect or suspected neurovascular uncoupling. These criteria resulted in a final cohort of 59 patients (35 women, mean age 29 years, SD 19 years), with a history of lesional or non-lesional epilepsy, brain tumors, or vascular malformations (demographics in Table 1).

**Table 1:** Demographics

Age	Range	10 to 72 yr
	Mean	29 +/− 16 yr
Sex	Female	35
	Male	24
Handedness	Left	12
	Right	45
	Unknown	2
Primary condition	Epilepsy/seizure	46
	AVM	6
	GBM	3
	Astrocytoma/oligoastrocytoma/mass	3
	Cavernous angioma	1
Location	L temporal	24
	R temporal	15
	L frontal	9
	R frontal	3
	L hemisphere	2
	Bitemporal	1
	L parietal	1
	L occipital	1
	Splenium of CC	1
	No records	2
Wada	No test	50
	Tested but no record	3
	L hemi	4
	L >> R	1
	R hemi	1
Complications	No surgery	19
	No complications	18
	No records postop	15
	Language deficits	7

Handedness as described in the original radiology fMRI report or the clinical record

*AVM* arteriovenous malformation, *GBM* glioblastoma, *CC* corpus callosum

For our analysis of postoperative outcomes, we included patients with postoperative MRI scans and postoperative neurological assessments documenting the presence or absence of new language deficits (*N* = 19). Examples of language deficits included “difficulties with word finding and dysarthria,” “receptive aphasia,” or “phonemic and semantic paraphasia.” Of this subgroup, 1 patient was excluded due to gross morphological changes in the brain that prevented co-registration of the postoperative resection mask with the preoperative activation maps. This resulted in a final postoperative outcome cohort of 18 patients (9 women, mean age 28 years, SD 13 years). Of these 18 patients, 6 had postoperative language deficits (4 women, mean age 28 years, SD 18 years).

For our subgroup analysis of patients with Wada testing, we included patients with records of preoperative Wada assessment (*N* = 9). Of this subgroup, 3 patients were excluded because either their Wada was reported as unsuccessful or the report of the testing was not available in the medical record. This resulted in a final Wada assessment cohort of 6 patients (2 women, mean age 28 years, SD 10 years).

### Imaging protocol

MRI data were collected on a 3.0 tesla GE 750 scanner (GE Healthcare, Chicago, IL) using an 8-channel head coil. Blood-oxygen-level-dependent (BOLD) data for fMRI tasks were acquired using a standard gradient-EPI sequence (4.7 mm slice thickness, 4.7 mm spacing, TE = 35 ms, 90° flip angle, using a 64 × 64 matrix, resulting in an in-plane resolution of 2 mm). All subjects performed 3 block design language paradigms: verb generation (VG), mental rhyming (MR), and passive listening (PL), as well as 1 non-language paradigm: auditory tones (AT), that served as a non-lateralizing control task. For the language fMRI paradigms (VG, MR, PL), 96 slices were acquired, with a 4000 ms TR. For the control fMRI paradigm (AT), 60 slices were acquired, with a 3000 ms TR. To allow for registration into MNI-152 standard space (2 mm resolution), a T1-weighted sagittal 3D FSPGR sequence was also acquired for each participant (256 × 256 matrix, 1 mm^3^ isotropic voxel size, TE = 2.98 ms, TR = 7852 ms, 13° flip angle).

### Behavioral protocol

All subjects performed 3 language paradigms: verb generation (VG), mental rhyming (MR), and passive listening (PL). Each consisted of 24 s of a control task followed by 24 s of the active task repeated for 8 cycles, for a total task duration of 6 min and 24 s. VG and MR tasks were covertly performed in response to visual stimuli back-projected on a screen visible to the subject through a mirror mounted on the head coil. On VG active blocks, subjects viewed a noun every 2–3 s while instructed to “think of a verb that goes with the noun.” On MR active blocks, subjects viewed a pair of words every 2–3 s while instructed to “decide if the words rhyme by thinking yes or no.” During control blocks, similarly complex non-linguistic symbols were presented, and subjects were instructed to simply look at the symbols. On PL active blocks, subjects were instructed to “follow along” with a narrative played through headphones. During control blocks, the narrative was played backward, and subjects were instructed to relax. Lastly, a non-language paradigm, auditory tones (AT), was also performed by all subjects. AT task blocks were 15 (rather than 24) s long and were repeated for 6 (rather than 8) cycles, for a total task duration of 3 min. On AT active blocks, subjects passively listened to binaural tones of differing frequency presented through headphones while instructed to relax. During control blocks, no stimulation occurred.

### Clinical analysis pipeline

During the original clinical testing sessions, GE BrainWave imaging software [8] generated a general linear model (GLM)- based statistical parametric (or contrast) map showing voxels with greater BOLD activation during active (“ON”) compared to control (“OFF”) blocks of each task (Online Resource 1). Corresponding structural 3D FSPGR T1-weighted images were segmented to remove the scalp and skull. The fMRI volumes were aligned using automated image registration to minimize motion artifact and were smoothed with a Gaussian filter of FWHM 8 mm. The data were then analyzed on a voxel-wise basis using a GLM that incorporated motion parameters as nuisance regressors, which generated a T-statistic map reflecting the contrast between the ON and OFF task blocks. The variance due to within-run linear drift was accounted for by using a ramp function as a covariate of no interest. The method of Worsley and Friston was used to estimate the effective numbers of degrees of freedom to account for temporal autocorrelation due to the smoothness of the hemodynamic response [9]. Using this estimate for degrees of freedom, the T-map was converted to a Z-map. The adjusted Z threshold for *T*-value of 0.01 was obtained using the unified T-method of Worsley [10]. The activation maps were then registered to a structural MRI using automated image registration. The resulting structural matched activation maps were ultimately overlaid onto the unsegmented structural MRI.

**Fig. 1:**
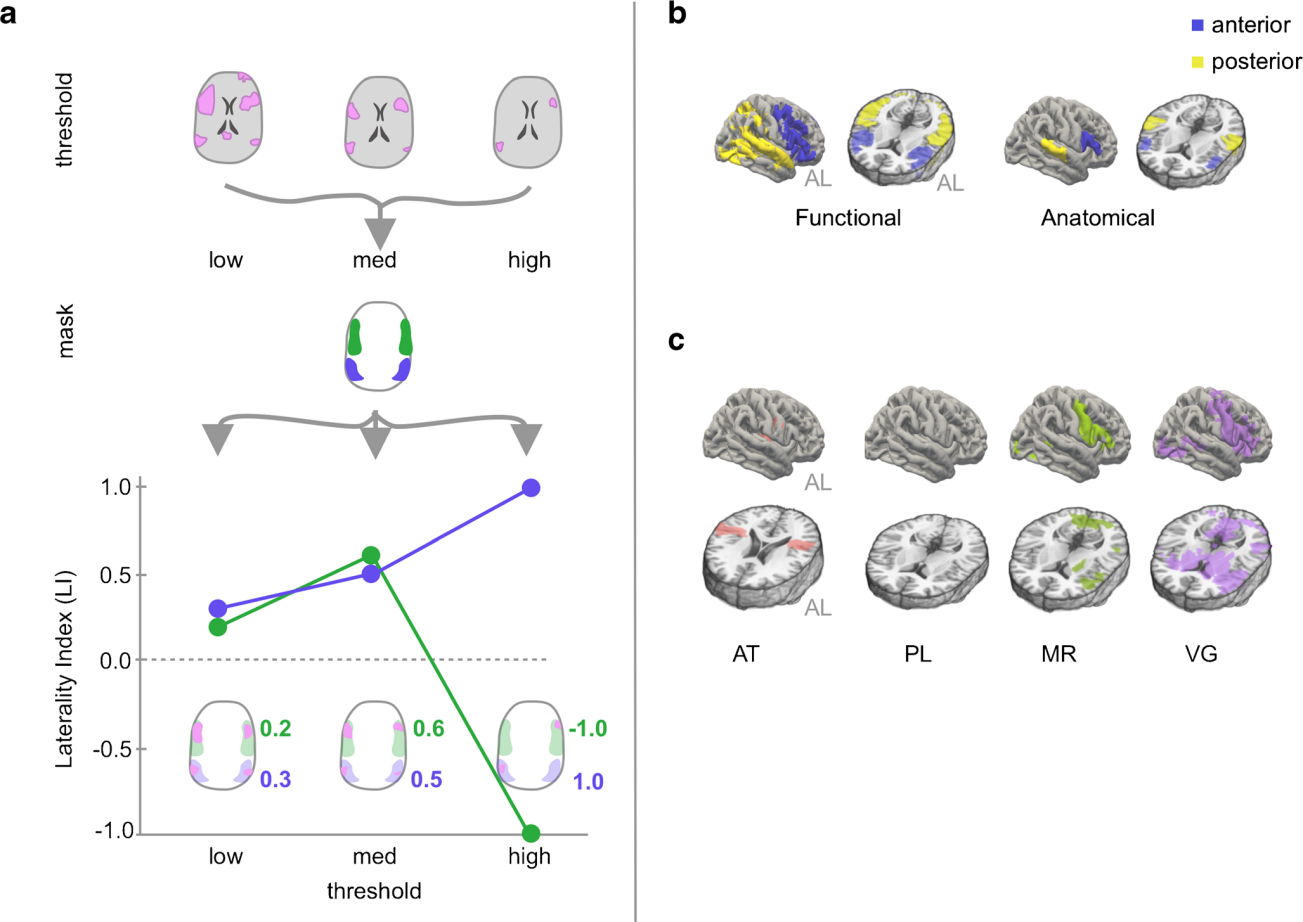
**a** For the same data, different activation thresholds (top), combined with different spatial masks (middle), can result in different laterality index (LI) results (bottom). For the spatial masks, green and blue represent masks for Broca’s and Wernicke’s areas, respectively. In the line plot, at low thresholds, LIs for the green and blue masks are similar, but at high thresholds, LIs for green become negative, while those for blue become more positive. **b** Functionally and anatomically defined language masks projected onto a pial surface. The functional mask is derived from the Neurosynth functional neuroimaging meta-analysis platform, thresholded and *p*-corrected ≤ 0.01; the anatomical mask is derived from the Harvard–Oxford cortical atlas. **c** Group-level activation maps for each of the 4 fMRI tasks (1 control task and 3 language tasks) show voxels that have higher  activation levels during the active blocks compared to the control blocks using a GLM and *p*-corrected ≤ 0.01 after TFCE. AL anterior-left brain orientation, AT auditory tones, PL passive listening, MR mental rhyming, VG verb generation

At the time of original testing, the neuroradiologist who performed the clinical testing reviewed these activation maps while manually adjusting the threshold to arrive at the clinical assessment of language laterality, which was then documented in the original radiology report. In addition, burnt-in-pixel (BIP) maps, which outlined areas of activation that exceeded a fixed statistical threshold (z-stat = 4.5), were automatically generated and overlaid on the structural images. These BIP maps provided a common quantitative output for each task/patient and were exported for intraoperative guidance.

### Research analysis pipeline

For each subject and task, we reprocessed the raw BOLD data offline using the FSL analysis package (FMRIB, Oxford, UK, version 5.0.8) [11] to allow for both subject-level and group-level analyses. For the subject-level analysis, we used FLIRT [12, 13] to register the functional data to the high-resolution structural image and the high-resolution structural to Montreal Neurological Institute (MNI-152) space. The following prestatistics processing was applied to the BOLD data: motion correction using MCFLIRT [13]; non-brain removal using BET [14]; spatial smoothing using a Gaussian filter of FWHM 5 mm; grand-mean intensity normalization of the entire 4D dataset by a single multiplicative factor; high-pass temporal filtering (Gaussian-weighted least-squares straight line fitting, with sigma = 50.0 s). When directly comparing the FSL and BrainWave pipeline outputs, the Gaussian kernel was modified to match the BrainWave filter of FWHM 8 mm. We carried out time series statistical analysis using FILM with local autocorrelation correction [15]. The time series model included regressors for the control and active blocks (24 s duration for language tasks, 15 s duration for the auditory task), temporal derivatives and standard motion parameters using a double gamma hemodynamic response convolution function. All group-level GLM analyses were carried out using randomise, a nonparametric permutation inference tool [16], to apply threshold-free cluster enhancement. The resulting contrast maps were thresholded at *p*-corrected of either ≤ 0.01 or 0.05, as stated.

### Quantifying the laterality of activation

We based our assessments of laterality on the laterality index (LI), a metric comparing the relative number of active voxels in the left and right hemispheres [17].

$$\mathrm{LI}=\frac{\mathrm{LH}-\mathrm{RH}}{\mathrm{LH}+\mathrm{RH}}$$

LH and RH represent the number of voxels within the left and right hemispheres that exceed the activation threshold *and* fall within a specified mask. LIs vary from −1 (fully right-dominant) to +1 (fully left-dominant). In practice, we computed LIs using SPM’s LI toolbox (SPM8, 2009) [18].

In general, LI results depend crucially on both thresholds and masks, as illustrated in Fig. 1a [19]. Specifically, to compute LIs, activation maps are first “thresholded” at a particular statistical level and then “masked” to only count voxels within a specified region of interest. In Fig. 1a, green and blue represent masks for Broca’s and Wernicke’s areas, respectively. In the plot of resulting LIs, we see that for the same underlying activation, low statistical thresholds produced LIs that were similar for Broca’s and Wernicke’s masks, but higher thresholds produced divergent results, with LIs for Broca’s reversing from positive to negative.

### Steps in developing an “optimized LI”

To address these concerns, we developed an “optimized LI.” First, we compared the ability of different language masks to capture language activations in our population. Second, we systematically explored how LIs computed using these masks varied as a function of activation threshold and used this approach to test the relative efficacy of different language tasks in determining language dominance at both the subject and (using percentile thresholds) population level. Third, we defined the optimal LI threshold as the percentile threshold that maximized the correlation between the results of our LI approach and those of the original clinical radiology reports. Below, we outline in detail the methods associated with each of these 3 steps.

#### Step 1: Comparing candidate regional language masks

We compared anatomically and functionally derived language masks for determining language laterality. The anatomical mask consisted of bilateral cortical regions corresponding to Broca’s and Wernicke’s language areas derived from the Harvard–Oxford atlas [20]. Specifically, Broca’s area was defined as the inferior frontal gyrus, pars triangularis, and pars opercularis, and Wernicke’s area as the planum temporale and adjacent superior temporal gyrus [3]. The functional mask was generated using the Neurosynth meta-analytic package from an analysis of approximately 1100 published fMRI studies and 43,000 activations based on the term “language” with no additional thresholding applied [21]. The functional mask was created by reflecting the left-hemisphere meta-activation map across midline and excluding non-cortical brain regions (Fig. 1c). For each mask, we defined anterior and posterior submasks, with the anterior submask comprising the region within the anatomic frontal lobe.

To compare each mask’s ability to capture language-related activation in our clinical population, we used the Jaccard Index [22, 23] to measure the spatial overlap between candidate language masks and FSL-derived group-level GLM task activation maps:

$$\mathrm{J}\left(\mathrm{M},\mathrm{A}\right)=\frac{\mid A\cap M\mid }{\mid A\bigcup M\mid }$$

where *M* represents voxels within the specific language mask and *A* represents voxels within the population activation map exceeding a specified statistical threshold.

#### Step 2: Assessing relative task performance through variable threshold LIs

At the subject level, we first generated task-specific activation maps. We then adopted an approach that walks a middle ground between threshold-dependent and threshold-independent methods to determine how LIs changed as a function of the threshold [7]. Specifically, we used a variable-threshold approach that divided each subject’s distribution of activation across all tasks and masks into equally populated percentile bins (excluding the top percentile of values as outliers). For each mask and task, we then calculated the LI for voxels whose activation *z*-statistic falls within each of these subject-specific percentile bins. This process had the effect of minimizing the influence of outliers and the particular shape of each subject’s underlying distribution of activations on LI calculations: the resulting LIs were expressed not in terms of raw *z*-statistics but in terms of percentile-based (“normalized”) values.

To evaluate how LIs varied as a function of threshold at the population level, we simply averaged these percentile-based LIs across subjects at each percentile bin and displayed results for all percentiles that have data from ≥ 10 subjects. This allowed us to compare LIs between masks and tasks across the population. We validated these task rankings using an independent, pairwise group-level GLM analysis that compared the activation between tasks. To address the possibility that tumor angiogenesis or shunting from vascular lesions might result in neurovascular uncoupling that could impact the reliability of the BOLD signal [24, 25] and thereby influence our results, we also performed the population-based LI analysis separately for patient subgroups with (*N*= 12) and without (*N*= 46) a diagnosis of mass or arteriovenous malformation (AVM).

#### Step 3: Estimating the optimal threshold

To determine the optimal LI threshold, we compared the results of our LI approach to those contained in the original clinical radiology reports. First, we converted the descriptive clinical assessments of laterality in the radiology reports into a numerical scale. Specifically, we relied only on the text of the reports themselves (i.e., blinded to the functional activation maps) and coded the strength of each patient’s language laterality that was recorded in the original report using the following scale:

1: Strong right-lateralized
2: Weak right-lateralized
3: Bilateral but predominantly right-lateralized
4: Bilateral
5: Bilateral but predominantly left-lateralized
6: Weak left-lateralized
7: Strong left-lateralized

The language laterality scores were coded from the original reports in the following manner: If the radiology report described the activation as lateralized and “strong,” “robust,” “consistent with lateralization,” or “hemisphere dominant,” the patient was scored as “strong (L- or R-) lateralized.” If the report described the activation as lateralized but “weak” or “to a lesser extent,” the patient was scored as “weak (L- or R-) lateralized.” If the report described the lateralization as “bilateral” without further qualification, then the patient was coded as “bilateral.” If lateralization was described as bilateral but with some asymmetry, then the patient was coded as “bilateral but predominantly (L- or R-) lateralized.”

For each patient, we separately coded strength of laterality reported for anterior-expressive and posterior-receptive language areas and used the average as the overall clinical assessment score. To find the optimal LI threshold, we then correlated the set of population LIs computed at each percentile of subject activation with these clinical assessment scores to determine the percentile threshold that maximized the correlation between clinical assessment scores and LI results across the population. In computing these correlations, we included each subject’s LIs from both VG and MR tasks (excluding PL due to its poor overall performance).

### Comparison to postoperative outcomes and Wada

For the subgroup of patients (*N* = 18) with available postoperative MRI and neurological follow-up that documented the presence or absence of language-related complications, we compare the resected volume to the masked and thresholded language maps used to compute the optimized LI for that patient to determine whether patients with higher overlap between the resected regions and language activation maps were more likely to experience postoperative language deficits. A neuroradiologist (LS) used the ITK-SNAP software package [26] to perform voxel-wise hand segmentations of the resection cavities (or lesion volumes in the case of Gamma Knife patients) on the structural FLAIR sequence from each patient’s postoperative MRI, and incorporated information from additional sequences as appropriate. We co-registered these FLAIR sequences with each patient’s masked and thresholded language activation map and lastly measured the volumetric overlap as the intersection between the resected ROI and language activation map (representative subject shown in Fig. 3c).

For the small subgroup of patients with preoperative Wada testing (*N* = 6), we compared our optimized LI results tothose of Wada testing. Five subjects had clear left or right hemisphere dominant language based on Wada and 1 patient had a recorded Wada assessment of L >> R hemisphere, which we coded as belonging to the left hemisphere dominant group.

### Statistical analysis

All statistical analyses were completed as described. FSL-derived activation maps and Neurosynth masks were corrected using threshold-free cluster enhancement [16] and FDR, respectively, at *p* < 0.01. Group-level GLM-based contrasts of task activations were performed in a pairwise manner at a less stringent threshold of *p* < 0.05 to display activation from all 3 language tasks. Comparison of the overlap between the resected region and the optimally thresholded language activation map for patients with or without postoperative language complications was performed using the Wilcoxon rank-sum test due to the non-normative distributions of the two language outcome groups.

## Results

### Comparing functional and anatomical language masks

We compared the ability of functional and anatomical masks to capture language-related activation in our clinical population using the Jaccard Index (a measure robust to differences in the absolute size of the sets being compared) to quantify overlap between each mask/submask (Fig. 1b) and our FSL group-level GLM-derived task activation maps (Fig. 1c). Compared to the anatomical masks, the functional mask and its subcomponents showed greater overlap with these group-level activation maps for both MR and VG tasks (JI of 0.1 vs. 0.09 and 0.26 vs. 0.06, respectively). For PL, no voxels survived TFCE thresholds of *p* ≤ 0.01 or *p* ≤ 0.05 (Online Resource 2).

### Quantifying laterality as a function of threshold

**Fig. 2:**
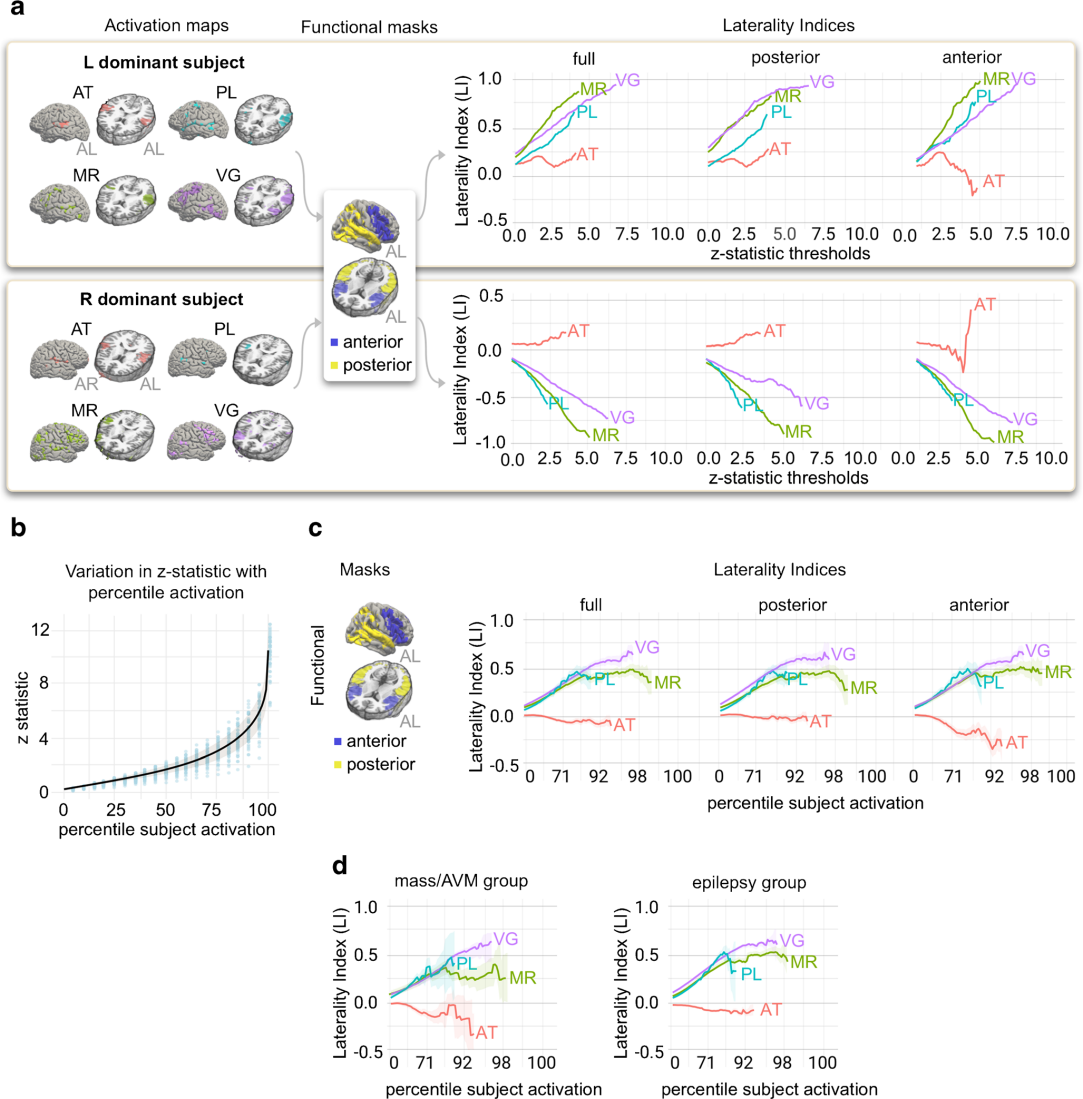
**a** For representative left (upper) and right (lower) language dominant subjects, activation maps for each fMRI task show voxels that have more activation during the active blocks compared to the control blocks. Maps are thresholded at *z*-statistics of 3.5 and 3.0 for the left- and right-dominant subjects, respectively. Graphs show how the laterality index (LI) for each subject varies as a function of increasing *z*-statistic threshold for each of the 4 tasks and the full or partial functional masks. **b** Relationship between *z*-statistics and percentile subject activation. At each percentile activation, blue circles plot the range of *z*-statistics observed across the population of 59 subjects. For the population, the fitted black curve and shaded gray region show how the average +/− SD *z*-statistic varies as a function of percentiles of subject activation. **c** For the functional mask, graphs show how the average and standard error (shaded region) of the population laterality index vary as a function of percentile thresholds for each of the 4 tasks and the full or partial masks. **d** The 2 patient subgroups (patients with masses/AVMs and patients with epilepsy), show a similar pattern of change in the average laterality index as a function of percentile thresholds when compared to each other and the entire population (**c**). AL anterior-left brain orientation, AR anterior-right brain orientation, AT auditory tones, PL passive listening, MR mental rhyming, VG verb generation

Figure 2a (left panels) shows sagittal and axial task activation maps for representative subjects with left (top) and right (bottom) hemispheric language dominance. For each subject, the right-sided panels show how LI for each task varied as a function of linearly increasing the *z*-statistic threshold. For the full functional mask, and each subcomponent, the plots highlight how LI varied by threshold and task — for the left-dominant subject (top), VG produced larger LIs at higher thresholds when compared to the other tasks, while for the right-dominant subject (bottom), MR produced larger LIs at higher thresholds. The plots also demonstrate how maximum activation varied both between subjects and between different tasks/masks for a single subject.

To facilitate population-level comparisons, we computed LIs as a function of percentile thresholds of each subject’s voxel-wise activation across all tasks. Using percentiles allowed us to compare across tasks and subjects without assumptions about the underlying distribution of activations in individual subjects. Figure 2b shows the range of *z*-statistics across the 59 subjects corresponding to these percentiles of subject activation. This plot highlights (1) the variability in individual subject activations at each percentile and (2) the exponential shape of the overall relationship between *z*-statistics and percentiles of activation. Subsequent population-level analyses expressed activations as percentiles of individual subject activation on a log axis.

Figure 2c shows results of a population-level analysis analogous to the subject-level analysis in Fig. 2a. For the full functional mask and each subcomponent, for each task we plotted the average and standard error (shaded region) of the LIs across the population at each percentile threshold. Figure 2d shows results of the same population-level analysis applied separately to the subgroup of patients whose primary diagnosis was AVM or mass and the subgroup whose primary diagnosis was epilepsy. For the population as a whole and for both subgroups, we saw clear differences in language task performance; in particular, at thresholds above the 90th percentile, VG produces greater average laterality indices than MR, with both VG and MR outperforming PL. An analogous population-level plot created using the anatomic masks can be found in Online Resource 3.

### Group-level GLM-based comparison of language tasks

**Fig. 3:**
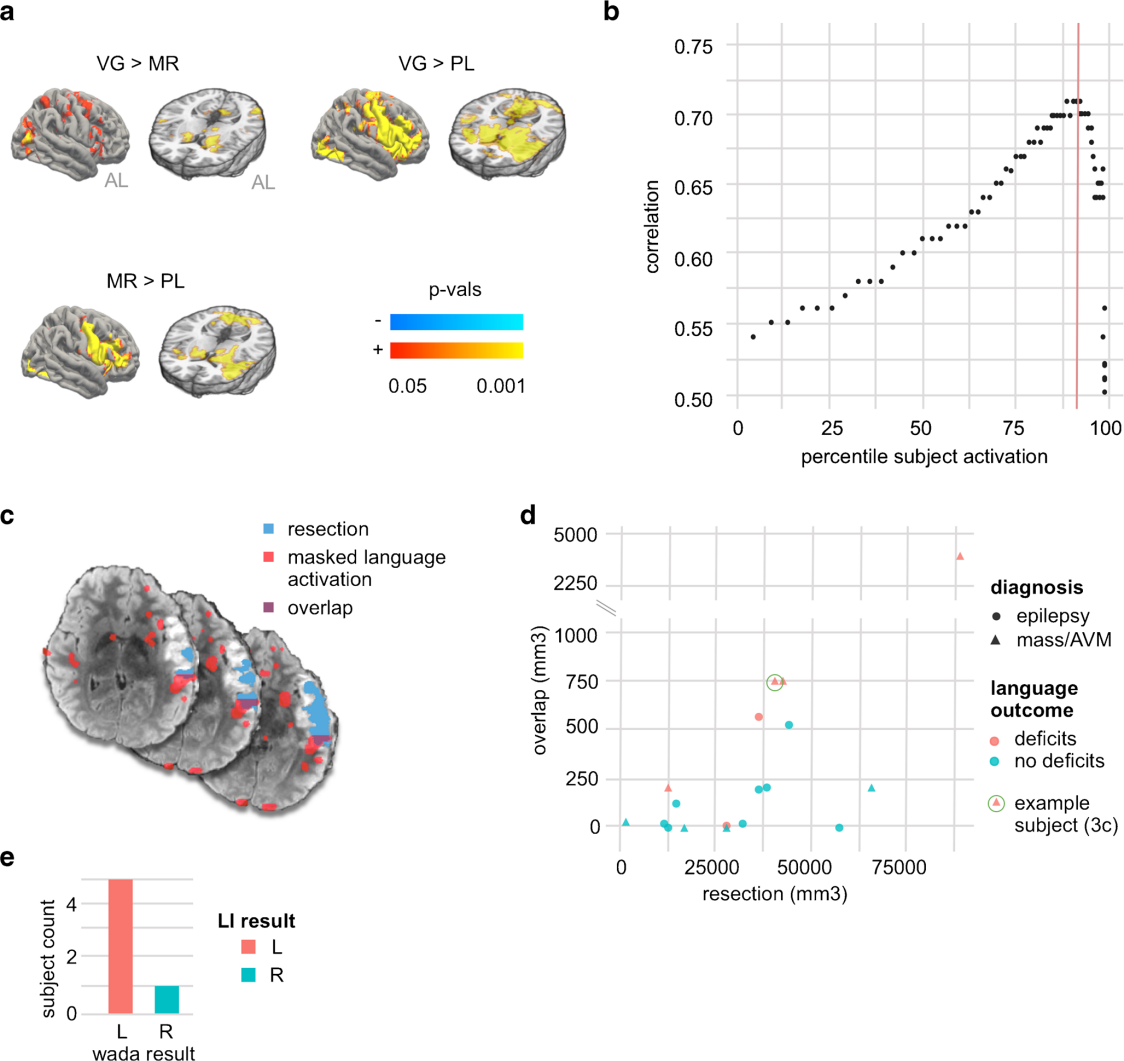
**a** Group-level contrast maps show areas that are differentially activated between different language tasks. Each contrast map represents results from a pairwise group-level analysis between the two listed tasks, thresholded at *p*-corrected ≤ 0.05 after TFCE. **b** Correlation between clinical scores and laterality indices computed at percentiles of subject activation for MR and VG tasks. Vertical red line highlights the percentile yielding the highest correlation (92nd percentile, corresponding to a correlation coefficient of 0.71). **c** For a representative subject with postoperative language deficits, the overlap (purple) is shown between the resection mask (blue) and the optimally thresholded language map (red). **d** A comparison of the overlap between the resection mask and the optimally-thresholded language map (*y*-axis) as a function of the volume of the resection mask (*x*-axis) is shown separately for patients with (red) and without (blue) postoperative language deficits. Overlap is shown separately for subjects with diagnoses of mass/AVM (filled triangle) or epilepsy (filled circle); the example subject shown in **c** is highlighted with a green circle. **e** Wada assessments are compared to LI results for laterality (left or right hemisphere). AL anterior-left brain orientation, AT auditory tones, PL passive listening, MR mental rhyming, VG verb generation, LI laterality index

The results of our LI approach suggested a clear task ranking: the VG task, analyzed using the functional mask, performed best in lateralizing both anterior and posterior language function in our presurgical clinical population. We validated this task ranking through an independent analysis that does not depend on laterality metrics. Specifically, using FSL, we performed a group-level pairwise GLM analysis contrasting activation between tasks for each subject (Fig. 3a). In accordance with our LI-based results, when directly compared at the population level, we arrived at the same task rankings: VG produced significantly more activation than MR within both anterior and posterior language areas, and both VG and MR outperformed PL.

### Comparison to clinical assessments

Having demonstrated which tasks performed best in determining language laterality at the population level across a range of percentile-based thresholds, we asked whether a specific percentile threshold maximized the correlation between our results and the laterality assessments made at the time of original clinical testing and documented in each patient’s original radiology report. To do this, we first scored the laterality assessments from the original radiology reports (see “Methods”). Across all subjects, we then correlated the set of LIs computed at each percentile of subject activation with these clinical scores. Figure 3b shows that the correlation between LIs and clinical scores peaks at a percentile threshold of 92 (*R* = 0.71). At this threshold, the *z*-statistic for individual participants ranged from 4 to 10 (Online Resource 4). In contrast, when we used a fixed *z*-statistic of 4.5 to threshold each subject’s activation maps (as is the case for the BIP maps generated by BrainWave software), we found a much lower correlation between resulting LIs and clinical scores (*R* = 0.56).

### Comparison of results to postoperative outcomes and Wada assessments

As an additional validation of our optimal LI results, we compared the masked and thresholded language activations to postoperative language and imaging outcomes and also compared LI laterality results with laterality derived from preoperative Wada testing. When comparing subject-level language activations to postoperative outcomes, we found significantly greater overlap between resected regions and language activations in subjects with postoperative language deficits compared to those without (Fig. 3d). This difference was present in both our entire population (*p* = 0.019) and in the subset of 8 subjects with a primary diagnosis of mass/AVM (*p* = 0.040). Furthermore, for the small subgroup of 6 patients with results from preoperative Wada testing, the laterality determined from our optimized LI-based analysis was in complete agreement with laterality derived from the Wada assessment (Fig. 3e).

## Discussion

Here, we have developed a quantitative framework for determining language laterality in clinical fMRI. Building on the concept of the laterality index (LI), the approach has two key elements: (1) an unbiased functional mask derived from a meta-analysis of fMRI language-related activations and (2) thresholding based on percentiles of (individual) subject-level activation. We tested the approach by using it to rank the different language tasks used in our population, validating our task hierarchy through an independent population-level pairwise GLM analysis. Lastly, we estimated the optimal percentile threshold to use for our LI measures by finding the threshold that maximized correlations with the clinical assessments of laterality made at the time of original testing and documented in the original radiology report. While they cannot be considered “ground truth,” these assessments do reflect current best clinical practice in the field. Furthermore, where available, we validated our novel automated approach through comparison to postoperative language outcomes and preoperative Wada assessments.

There is a growing consensus that regional laterality indices are more appropriate for assessing language dominance than holo-hemispheric approaches [7]. However, what region (or regions) to factor into such analyses remains unclear. Here, we showed that a “functional” regional mask derived from a meta-analysis of fMRI language-related activations captured language-task activation in a presurgical clinical population better than a mask that reflects the classic Wernicke–Lichtheim–Geschwind language model [27]. This result is perhaps unsurprising from a neuroscience perspective, where the limitations of the classical model are well established [3]; however, the classical model remains remarkably prevalent in clinical practice. More contemporary models of language processing emphasize parallel processing pathways [28, 29], or distributed language networks that interact dynamically with brain networks responsible for attention and cognitive control [4]. Importantly, we do not suggest that the particular functional mask we employed provides an ideal regional characterization of language function, only that it provides an objective, data-driven and operationally defined benchmark against which alternative approaches can be compared.

We used this functional mask to explore how activation thresholds influence quantitative measures of laterality derived from language task fMRI. Measures like the laterality index are appealing because they offer standardized — even automated — assessments of language laterality; however, they depend critically on the threshold applied to activation maps: thresholds too low can overemphasize weak and spurious activations, and thresholds too high can overemphasize surviving voxels that might not represent the underlying pattern of activation. Both threshold-dependent [30, 31] and threshold-independent [7, 32] methods have been developed to address the unreliability of fixed-threshold laterality measures, the latter weighing the contribution of voxels according to their statistical score. Each approach not only has certain advantages but also makes certain assumptions about the shape or weighting of the underlying distribution of activated voxels. Advanced statistical methods such as bootstrapping [33] can avoid such assumptions and improve the robustness of LI calculations but are much more computationally intensive. Here, we have taken a middle ground by developing a variable-threshold approach that minimizes the influence of outliers and the particular shape of the underlying distribution of activations on LI calculations by analyzing each subject’s distribution of activations in terms of (equally populated) percentile bins.

We applied this approach to compare the relative efficacy of a set of different tasks used to assess language laterality in our clinical population, validated our results through an independent population-based analysis, and determined the optimal percentile threshold to reproduce the original clinical assessments of language laterality in these same patients. Our analysis was necessarily limited to the 3 language tasks used routinely for preoperative language mapping at our institution and includes additional subgroup analyses for patients with a primary diagnosis of a mass or AVM to verify that our results are not confounded by the inclusion of subjects for whom tumor angiogenesis or shunting from vascular lesions might result in neurovascular uncoupling. Among these tasks, we found that verb generation produced the most robustly lateralized activation, a result we confirmed through a separate group-level GLM-based analysis of the data that compared the relative strength of activation between tasks across the population. We further found that correlations between LI-based laterality estimates and clinical assessments of laterality from the original radiology reports peaked at a narrow range of thresholds centered around the 92nd percentile of subject activation. Interestingly, at this percentile-based threshold, absolute activation levels (voxelwise *z*-statistics) in individual subjects varied over a wide range. This is important because many clinical systems, such as the BrainWave system that we use in our clinical practice, threshold activation maps at a fixed *z*-statistic. Our results suggest that relative (e.g., percentile) rather than fixed thresholds would improve the accuracy of such systems.

Somewhat surprisingly, the two other language tasks that we tested—mental rhyming and passive listening—provided little added benefit with respect to determining language laterality in our population. Prior studies have compared the relative efficacy of different language tasks in healthy controls [34], or in specific clinical populations [31, 32, 35], and we are not suggesting that the best performing task among the limited set that we tested is optimal for language mapping more generally. However, our results do suggest that the clinical fMRI protocol currently used at our institution could be shortened without negatively impacting its ability to lateralize language function, and that verb generation might serve as a benchmark against which to test the efficacy of other candidate tasks or of approaches that use resting-state fMRI for mapping language networks [36]. Other language tasks, such as sentence completion and silent word generation, which emphasize expressive vs. receptive language function, were recently used by Agarwal and colleagues to investigate within-subject reproducibility/repeatability of lateralization indices in brain tumor patients between successive scans in a single scanning session [37]. They found that silent word generation produced higher LI repeatability within Broca’s area while sentence completion produced higher LI repeatability within Wernicke’s area. These results suggest that some combination of tasks that emphasize expressive and receptive language function might ultimately prove optimal in assessing language lateralization.

### Limitations

Our study has limitations. First, our functional mask is derived from fMRI data and is thereby limited by the temporal resolution of the BOLD signal and insensitive to the rich temporal dynamics of language processing [38, 39]. Knowledge of that temporal structure (from magnetoencephalography (MEG) or electrocorticography studies) may ultimately allow us to map language more completely in both the spatial and temporal domains through combined techniques such as simultaneous EEG-fMRI. Second, we do not know the “ground truth” with respect to the lateralization of language function in our subjects. Instead, we must appeal to consistency of findings when we compare the results of our optimized LI approach with: (1) a group-level GLM-based comparison of task activation (reflecting the standard in the research literature), (2) the laterality findings detailed in the original radiology reports (reflecting standard clinical practice) and (3) additional subgroup analyses of patients with available preoperative Wada testing (*N* = 6) or postoperative language outcomes (*N* = 18). However, these validation methods are obviously limited by the small number of patients for whom these data were available. Finally, our sample size is small (but comparable to recent studies) [35, 37] and heterogeneous (but representative of patients who undergo fMRI for presurgical language mapping), and our task comparisons are limited to the 3 specific language tasks used. Thus, the relevance of our results to other populations and tasks remains to be tested. Before a framework like ours could be integrated into clinical workflow, it would first need to be validated on additional datasets from other institutions, and ideally in populations whose language fMRI results can be compared to those from one or more complementary approaches such as Wada, MEG, or systematic intraoperative stimulation. To facilitate this effort, the code/pipeline used here is publicly available on Github (https://git.io/JkJBX). Ultimately, our hope is that other groups might apply a similar approach to their data so that we might eventually combine results across institutions to arrive at an objectively defined set of highest performing tasks and practices.

## Conclusion

In clinical practice, fMRI has become widely accepted as a noninvasive alternative to Wada testing and intraoperative electrical stimulation for lateralizing language function [1, 2]. While effective at lateralizing language, developing fMRI as a tool to localize language at the sub-hemispheric level in individual patients has proved challenging. This challenge stems in part from a lack of standardized protocols and analysis methods in the field which has made it difficult to compare results across patients and institutions [6]. Recently, Benjamin and colleagues showed that clinicians trained to use subject-specific thresholding and an expanded set of language areas in interpreting fMRI for language lateralization generated assessments that were more reliable and more concordant with results of Wada testing than those who used fixed activation thresholds and regions of interest based on the classical anatomical model of Broca and Wernicke [5]. Our results also support the use of an expanded network of language areas and individual subject thresholding. However, we further show that a standardized approach based on an unbiased functional map of language areas and percentile thresholding of subject activation can both reproduce specialized clinical interpretations at the individual subject level and also allow for the type of population-level analyses that could establish best practices. Combined with a centralized database of anonymized fMRI and patient outcomes data, such a framework might allow us to systematically explore whether fMRI can localize language at a more granular level, increase our basic understanding of language processing, and improve patient care by better predicting postoperative language outcomes.

## Supplementary Information

ESM 1 (PDF 53.1 kb)

ESM 2 (PDF 34.2 kb)

ESM 3 (PDF 2.35 mb)

ESM 4 (PDF 36.7 kb)
