# Supplementary figures and images for: A quantitative approach for measuring laterality in clinical fMRI for preoperative language mapping
Source: Neuroradiology. 2021 Mar 26;63(9):1489–500. doi: 10.1007/s00234-021-02685-z (PMC8376727; doi:10.1007/s00234-021-02685-z)

## Task Completion

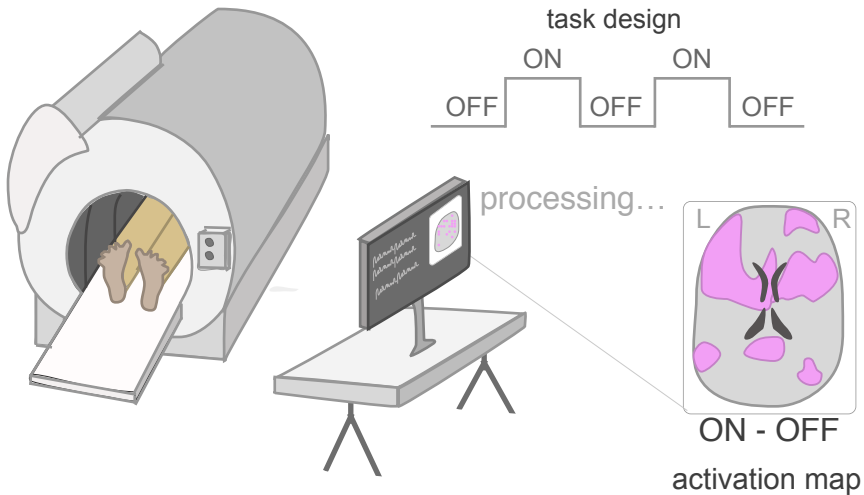

Supplement: Supplementary file 1 — (PDF 53.1 kb) [file 234_2021_2685_MOESM1_ESM.pdf]

## Population Average Laterality Indices

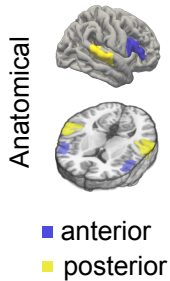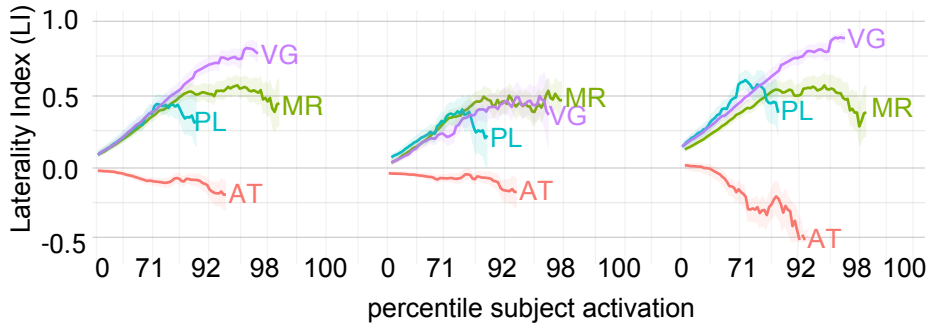

Supplement: Supplementary file 3 — (PDF 2.35 mb) [file 234_2021_2685_MOESM3_ESM.pdf]

## Z-statistical values at peak percentile of correlation to clinical assessments

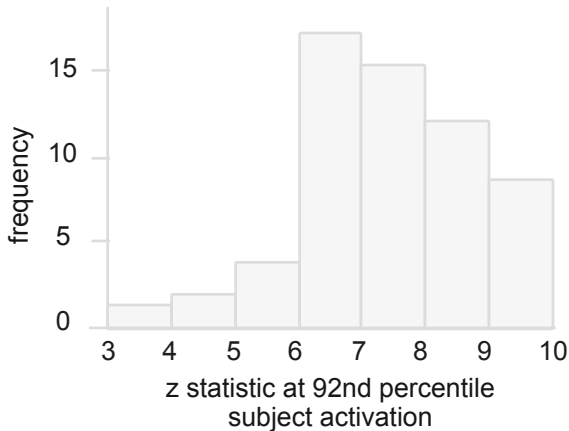

Supplement: Supplementary file 4 — (PDF 36.7 kb) [file 234_2021_2685_MOESM4_ESM.pdf]
