# Supplementary material for: A quantitative approach for measuring laterality in clinical fMRI for preoperative language mapping
Source: Neuroradiology. 2021 Mar 26;63(9):1489–500. doi: 10.1007/s00234-021-02685-z (PMC8376727; doi:10.1007/s00234-021-02685-z)
Supplement: Supplementary file 2 — (PDF 34.2 kb) [file 234_2021_2685_MOESM2_ESM.pdf]

Jaccard Index

$$\text{overlap} = \frac{\text{activation} \cap \text{mask}}{\text{activation} \cup \text{mask}}$$

Mask	AT	PL	MR	VG
Func full	0.07	0.00	0.10	0.26
Anat full	0.15	0.00	0.09	0.06
Func ant	0.01	0.00	0.17	0.42
Anat ant	0.00	0.00	0.20	0.10
Func post	0.12	0.00	0.05	0.13
Anat post	0.23	0.00	0.00	0.01
